# Development of a qPCR platform for quantification of the five bacteriophages within bacteriophage cocktail 2 (BFC2)

**DOI:** 10.1038/s41598-019-50461-0

**Published:** 2019-09-25

**Authors:** Hans Duyvejonck, Maya Merabishvili, Jean-Paul Pirnay, Daniel De Vos, Gilbert Verbeken, Jonas Van Belleghem, Tessa Gryp, Julie De Leenheer, Kelly Van der Borght, Leen Van Simaey, Stefan Vermeulen, Els Van Mechelen, Mario Vaneechoutte

**Affiliations:** 1 Laboratory Bacteriology Research (LBR), Department of Diagnostic Sciences, Faculty of Medicine and Health Sciences, Ghent University, Corneel Heymanslaan 10, 9000 Ghent, Belgium; 2 Department of Biosciences, Faculty of Education, Health and Social Work, University College Ghent, Keramiekstraat 80, 9000 Ghent, Belgium; 3 Laboratory for Molecular and Cellular Technology (LabMCT), Burn Wound Center, Queen Astrid Military Hospital, Bruynstraat 1, 1120 Brussels, Belgium; 4 The Eliava Institute of Bacteriophages, Microbiology and Virology, Gotua 3, Tbilisi, 0160 Georgia

**Keywords:** Bacteriophages, PCR-based techniques

## Abstract

To determine phage titers accurately, reproducibly and in a non-laborious and cost-effective manner, we describe the development of a qPCR platform for molecular quantification of five phages present in bacteriophage cocktail 2 (BFC2). We compared the performance of this molecular approach, with regard to quantification and reproducibility, with the standard culture-based double agar overlay method (DAO). We demonstrated that quantification of each of the five phages in BFC2 was possible by means of qPCR, without prior DNA extraction, but yields were significantly higher in comparison to DAO. Although DAO is assumed to provide an indication of the number of infective phage particles, whereas qPCR only provides information on the number of phage genomes, the difference in yield (qPCR/DAO ratio) was observed to be phage-dependent and appeared rather constant for all phages when analyzing different (freshly prepared) stocks of these phages. While DAO is necessary to determine sensitivity of clinical strains against phages in clinical applications, qPCR might be a valid alternative for rapid and reproducible quantification of freshly prepared stocks, after initial establishment of a correction factor towards DAO.

## Introduction

Given the widespread and increasing incidence of antibiotic resistance caused by the excessive use and misuse of antibiotics^1^, the development of new and alternative antibacterial therapies are being considered. The therapeutic use of bacteriophages is in this regard a topic of extensive research^1–3^. The number of phage therapy applications in Western medicine is rising^4–9^, e.g. the use of bacteriophages in the treatment of colistin-only-sensitive *Pseudomonas aeruginosa* septicaemia in a patient with acute kidney injury^5^ and use of phages against a multi-drug resistant isolate of *Acinetobacter baumannii* in a case of necrotizing pancreatitis^9^. All these phage application case reports and studies require phage titers to be determined accurately, reproducibly and ideally in a non-laborious and cost-effective manner in order to monitor the phage numbers during production of phage stocks as well as in different samples of the patients at different time points during the phage therapy application period.

At present, a culture-based double agar overlay (DAO) method is considered as a standard method for enumeration/quantification of bacteriophages. Phage concentrations are determined by mixing dilution series of a phage stock (in e.g. saline) with fresh cultures of a standardized concentration of the appropriate bacterial host, followed by plating of the mixtures, whereafter the number of individual plaques can be counted for some of the dilutions. This method was first described by Gratia^10^ and has been described more extensively recently^11,12^.

Because the DAO method is laborious, while its efficiency depends on several different factors such as plaque morphology, bacterial host, medium used, methods that are less time-consuming, that are possibly more reproducible, that require less hands on time and that could be automated, are warranted. In order to achieve this purpose, quantitative PCR (qPCR) has been shown to be a valuable approach^13–17^.

Here, we describe the design and applicability of a cohort of primers for the accurate quantification by means of qPCR of the five phages present in bacteriophage cocktail 2 (BFC2), which was developed as a therapeutic phage cocktail active against clinical strains of *Staphylococcus aureus, Pseudomonas aeruginosa and Acinetobacter baumannii*^12,18^. Workload, quantification results and reproducibility of both methods, DAO and qPCR, have been evaluated.

## Materials and Methods

### *In silico* primer design

Bacteriophage genome sequences (Table 1) were split in bins of 50.000 bases. A primer-BLAST was run (www.ncbi.nlm.nih.gov/tools/primer-blast) with the following parameters: PCR product size = 70–150; Minimal melting temperature (Tm) = 58 °C; Optimal Tm = 60 °C; Maximal Tm = 62 °C; Maximal Tm difference between primers = 2 °C; primer length (bp): minimal = 15 – optimal 20 – maximal 25; maximal GC in 5 last bases at 3′ end = 2; maximal identical bases at 3′ end <4; primer GC content: minimal 30% – optimal 50% – maximal 80%; and no T at 3′ end of primers. Primers were BLAST using the algorithm for short nearly exact matches against the nr/nt database. Primers were analyzed *in silico* using DINAMelt for homo- or hetero-dimer formation (unafold.rna.albany.edu). The amplicons were analyzed using mFold for investigating possible secondary structures. All ΔG negative circular structure plots were investigated manually. For primerBLAST, DINAMelt and mFold all concentrations of monovalent, respectively divalent cations were set to 50 mM and 3 mM. Tm’s were adapted for each calculation. The primer pairs and amplicons with the least homo- or hetero-dimer formation and secondary structures were selected.

**Table 1:** Phage designation, bacterial host, phage family, genome size and NCBI genome accession number of each bacteriophage present in BFC2.

Bacteriophage	Bacterial host	Family	Genome size (bp)	Accession number
Acibel004	*Acinetobacter baumannii*	Myoviridae	99730	NC_025462.1
Acibel007	*Acinetobacter baumannii*	Podoviridae	42654	NC_025457.1
PNM	*Pseudomonas aeruginosa*	Podoviridae	42721	Unpublished data
14/1	*Pseudomonas aeruginosa*	Myoviridae	66235	FM897211.1
ISP	*Staphylococcus aureus*	Myoviridae	138339	FR852584.1

### Production of bacteriophage stocks

Bacteriophage stocks were prepared as described previously^12^, using the double agar overlay plaque titration (DAO) method with minor modifications. For each phage and host, the ratio that resulted in maximal webbing was determined. In order to produce a sufficient volume of fresh crude phage stock, the mixture of phages and bacteria was made multiple times at once in separate sterile 14 ml polypropylene round-bottom tubes, whereafter 3.5 ml of liquid lysogeny broth (LB broth, VWR Chemicals, Leuven, Belgium), cooled to 40 °C, was added. The LB broth contained 0.6% (w/v) Bacto agar (Becton Dickinson, Erembodegem, Belgium), except for phage PNM (large plaques) for which 0.8% (w/v) Bacto agar was used, in order to limit the size of the PNM plaques. The mixture was plated onto a 90 mm diameter Petri dish filled with a bottom layer of LB agar and incubated at 32 °C for 18–24 h. Subsequently, 500 µl of chloroform (Sigma Aldrich, Steinheim, Germany) was added to the inverted lids of the Petri dishes and the inverted plates were further incubated on top of the inverted lids at 4 °C for 1 h. Thereafter, the top agar was scraped off using a sterile L-shaped rod and transferred to a sterile 50 ml centrifuge tube (Beckman Coulter, Brea, CA). The mixture was first centrifuged for 20 min at 6000 × *g*. Then the supernatant was aspirated using a sterile 20 ml syringe with a 22 G × 2” sterile needle and passed through a 0.45 µm membrane filter. The filtered supernatant was then centrifuged for 1 h at 35000 × *g*. Finally, the pellet was resuspended in saline in order to obtain a crude bacteriophage stock, used to develop the qPCR platform and to compare quantification by means of DAO and qPCR.

### Double agar overlay method for bacteriophage titration

The concentration, in plaque forming units/ml (pfu/ml), of each bacteriophage stock was determined by the standard method, i.e. the DAO method, performed on a tenfold serial dilution (10^0^ to 10^−12^) of the phage stock, as described previously^10,11,19^ and as adapted by Merabishvili *et al*.^12^. Dilutions were performed in saline to maintain phage stability and mixed together with their bacterial host and semi-solid LB agar before plating. To calculate the original bacteriophage concentration, plates with distinguishable homogeneous plaques were counted. Taking into account the dilution and the volume used for plate inoculation, the mean number of plaques was used to calculate the concentration of pfu/ml of the original stock.

### Isolation of DNA

DNA was extracted from all phages using the Purelink Viral RNA/DNA Mini Kit (Invitrogen, Carlsbad, California), following pretreatment with 4 µg of DNase I (STEMCELL Technologies, Grenoble, France) and 20 µg of RNase A (Invitrogen) of 200 µl of crude bacteriophage stock for the removal of bacterial nucleic acids. A volume of 100 µl of nucleic acids was eluted. DNA extracts were used to set up a calibration curve in a qPCR. For gradient PCR and qPCR on unknown crude bacteriophage stocks a hundredfold dilution was sufficient for quantification, and hence a preceding DNA extraction was not necessary.

### DNA yield and purity assessment

The yield and purity of isolated DNA was measured by a NanoDrop spectrophotometer ND-1000, software version 3.3.0 (ThermoFisher Scientific, Merelbeke, Belgium). The purity was assessed by means of the A_260 nm_/A_280 nm_ ratio.

### Calibration curve

To compensate for the reduction of the volume during the isolation of DNA (i.e., from 200 µl at the start to 100 µl at elution), the obtained concentration (ng/µl) measured with NanoDrop was divided by two. Subsequently, the unit of concentration for each phage DNA extract was converted to ‘genomes/ml’ by dividing the measured DNA concentration by the molecular weight of each phage genome. The molecular weight is calculated for each individual phage on the basis of the genome size, percentage of GC and AT, and the molecular weight of GC and AT. Starting from the phage DNA stock of each of the five phages, tenfold serial dilution series were prepared in HPLC grade water and added as target to the qPCR assay. Finally, the calibration curve was constructed using the obtained Cq values in the y-axis and logarithmic concentrations (genomes/ml) in the x-axis.

$$\begin{array}{ccc}MW\,genome\,(g/genome) & = & \frac{(GC\,bp\,number\times 618.4\,g/mol)+(AT\,bp\,number\times 617.4\,g/mol)}{Number\,of\,Avogadro}\\ Concentration\,(genomes/ml) & = & \frac{Concentration\,Nanodrop\,(g/ml)}{MW\,genome\,(g/genome)}\end{array}$$

### Gradient PCR followed by agarose gel electrophoresis

Gradient PCR was used to primarily evaluate the specificity of the designed primer pairs, and to determine the optimal annealing temperature for each qPCR assay. Every PCR reaction contained 200 nM of each primer, 12.5 µl of 2x Faststart PCR Master (Roche, Brussels, Belgium), 2.5 µl of a 10^−2^ dilution of crude bacteriophage stock (PCR template) and 9.5 µl RNase-free water for a total reaction volume of 25 µl. Dilutions of the phage template were performed in HPLC-grade water to maintain salt concentrations for reproducible PCR conditions. Amplification was carried out on a Veriti 96-Well Thermal Cycler (ThermoFischer Scientific) with the following program: pre-incubation for 5 min at 95 °C and amplification for 45 cycles of 30 s at 95 °C, 30 s at different annealing temperatures between 53 and 63 °C and 1 min at 72 °C, followed by a final elongation step of 5 min at 72 °C. Subsequently, 6.25 µl of every PCR product mixed with loading dye (ratio 4:1) was loaded onto a 1.5% agarose gel, containing EtBr, prepared in 1x TAE buffer. After electrophoresis in 0.5x TAE buffer the EtBr stained DNA bands were visualized using the Chemidoc XRS + gel imaging system (Bio-Rad, Eke, Belgium).

### qPCR

Different parameters were evaluated during the development of each qPCR assay, such as annealing temperature, primer and MgCl_2_ concentration and primer specificity. Results were analyzed and quantified with the standard LightCycler 480 Software, version 1.5 (Roche). Two µl of a hundred- or thousandfold dilution of the crude bacteriophage stock (qPCR template) was added to a total volume of 10 µl LightCycler 480 HRM master mix (Roche) containing 0.05, 0.10, 0.15 or 0.2 µM of each primer, 2.0 or 3.0 mM MgCl_2_ and High Resolution Master. Dilutions of the phage template were performed in HPLC-grade water to maintain salt concentrations for reproducible PCR conditions. Amplification was carried out on a LightCycler 480 (Roche), using the following program: pre-incubation for 5 min at 95 °C and amplification for 35 cycles of 10 s at 95 °C, 15 s at different annealing temperatures between 59 and 63 °C and 15 s at 72 °C, after which a high resolution melting curve was generated, using the following protocol: 1 min at 95 °C, 1 min at 40 °C, 1 s at 60 °C, followed by a gradual increase in temperature from 60 °C to 97 °C, using a ramp rate of 0.04 °C per s, with one measurement per 1.665 s.

### Data collection and statistical analysis

Culture-based quantification data were determined by manual plaque counting, after which the number of plaques was converted to concentrations (pfu/ml) taking into account the volume plated and the dilution factor. qPCR analysis resulted in Cq-values which were converted to concentrations (genomes/ml) using a calibration curve. Data were all log transformed prior to statistical analysis. Statistical analyses was performed by IBM SPSS Statistics for Windows, version 25 (IBM, Armonk, N.Y.) and graphics were made using SPSS or Excel. The reproducibility of each method was assessed with the coefficient of variation (C_v_ %). Values below 20% were considered to be indicative for low variability. All samples (for DAO and qPCR) from the same stock, and phage, were considered as paired samples and every phage was analyzed separately. This way, the paired Student t-test (for normally distributed data) or Wilcoxon test (for not normally distributed data) were used to compare both methods in quantification of phages. Intra- and inter-operator variability were evaluated by either the Linear mixed model (for normally distributed data) or a Friedman test (for not normally distributed data). P-values below 0.05 indicated that the obtained differences were statistically significant.

## Results

### Study set up

Initially primer pairs for quantitative amplification of DNA from each phage of bacteriophage cocktail 2 (BFC2) were designed by application of bioinformatics tools. The efficiency and specificity of each *in silico* designed primer pair was evaluated by performing qPCR on all five different bacteriophage stocks. For each phage, the most specific and efficient primer pair was selected. Subsequently, the qPCR platform was evaluated for its ability to accurately and rapidly determine the amount of each bacteriophage of BFC2, in comparison with the current gold standard method of double agar overlay (DAO).

### *In silico* primer design

A total of 23 primer pairs were withheld in the *in silico* design out of which four were further selected for Acibel004, two for Acibel007, two for PNM, eight for 14/1 and seven for ISP (Supplementary Table S1).

### Evaluation of primer efficiency and specificity

**Figure 1:**
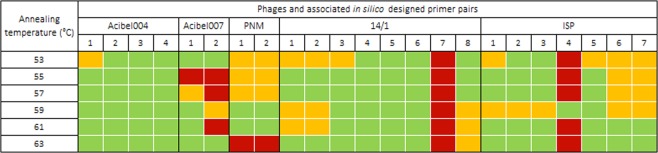
Primary evaluation of the *in silico* designed primer pairs at different annealing temperatures (53–63 °C) by means of gradient PCR and agarose gel electrophoresis. Specific amplification resulting in strong (green) or weak (orange) amplification, or aspecific amplification (red).

The primary evaluation with gradient PCR and agarose gel electrophoresis revealed that most designed primer pairs were specific, resulting in a single band amplicon with the expected length. Analysis of the intensity of the amplicons obtained at different annealing temperatures indicated that the optimal annealing temperature for the qPCR platform was between 59 and 63 °C (Fig. 1).

In further qPCR experiments, we compared the specificity and efficiency of using different annealing temperatures (59, 60, 61, 62 and 63 °C), MgCl_2_ concentrations (2 and 3 mM) and primer concentrations (0.05, 0.1, 0.15 and 0.2 µM) (Supplementary Fig. S1 shows the results for primer pair 1 of phage PNM). We observed that for each bacteriophage efficient primer pairs were found at an annealing temperature of 60 °C, a Mg^2+^ concentration of 3 mM and a primer concentration of 0.2 µM. Subsequently, the specificity of these efficient primer pairs was further investigated by evaluating the cross reactivity with other phages and bacterial hosts. Supplementary Fig. S2 shows the absence of cross reactivity for the primer pairs that were selected for each of the five phages. For each bacteriophage, only one primer pair was selected for further investigation (Indicated in Supplementary Table S1 with asterisk). Cq differences and % efficiency of each qPCR are given in Supplementary Fig. S3. Because the evaluation of the *in silico* designed primers was carried out with individual phages, whereas BFC2 consist of five phages, we further analyzed specificity and efficiency of the primers on a mixture of the five phages. Results indicated that each individual phage was accurately quantified, without cross reactivity of each of the primer pairs with any of the other four phages (data not shown). Limit of detection was between 2 and 20 phage genomes per PCR mixture, *i.e*., 10^3^–10^4^ phage genomes per ml. Whereas for DAO the limit of detection was between 10–20 pfu/ml.

### Comparison of phage quantification by means of direct qPCR and by means of culture

**Figure 2:**
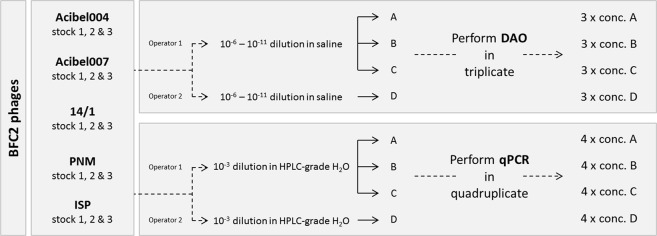
Experimental set-up for the quantitative and qualitative comparison of qPCR and DAO.

In order to determine to what extent quantification by means of direct qPCR – *i.e*., without preceding DNA extraction from the phages – correlated with that obtained by means of the culture-based DAO plaque assay, three stocks of each bacteriophage were produced (Fig. 2). For the DAO method, three complete assays were done on the same day, starting from the same bacterial suspension. Four separate titrations in saline were carried out for each phage stock. For every titration the different phage dilutions were plated out in triplicate. Plaques were counted and plaque numbers were converted to concentrations (pfu/ml). For the evaluation of the qPCR platform, a thousand-fold dilution in HPLC graded H_2_O of each stock was prepared four times. Subsequently, each dilution was run in quadruplicate on the qPCR platform. All dilutions, together with the standard curve, were carried out in a single thermal cycling run. With the aid of a calibration curve, the Cq values were converted to phage genomes/ml. Data of operators 1 and 2 were also used to assess the intra- and inter-operator variability of both methods.

**Figure 3:**
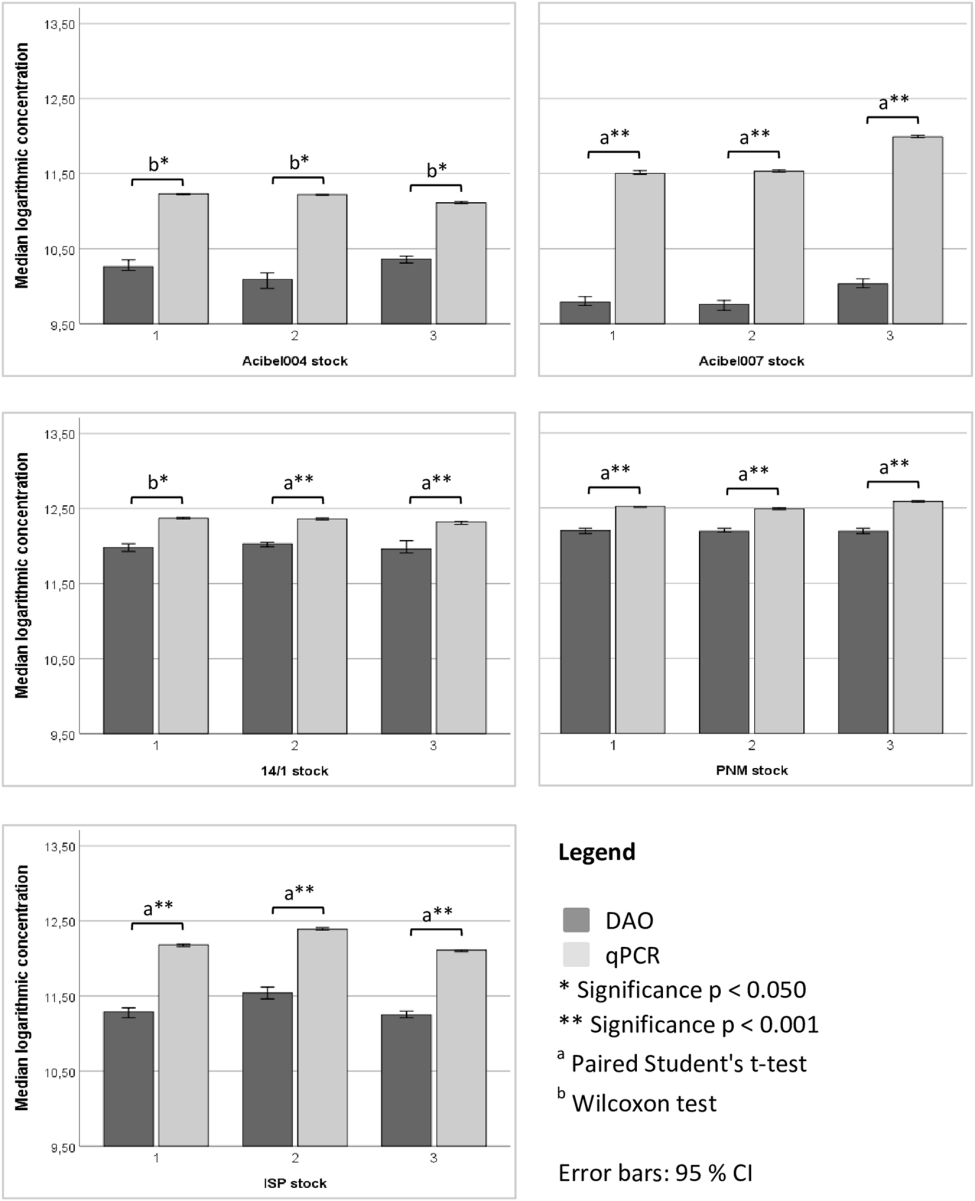
Estimated amount of phages detected by qPCR and DAO. SPSS statistical analysis was done using the Paired Student’s t-test (a) or Wilcoxon test (b). Data presented as median logarithmic values with 95% CI.

**Figure 4:**
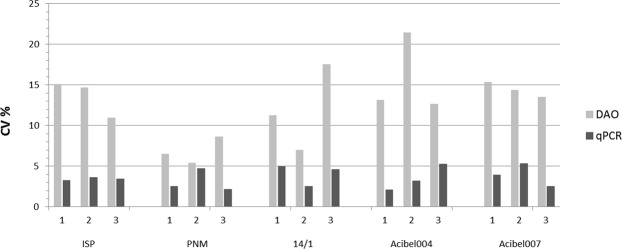
Comparison of the reproducibility of quantification by means of qPCR and DAO on the basis of the coefficient of variation (CV %). The mean values (A, B and C) of each stock of operator 1 were taken into account.

qPCR always yielded a significantly higher estimate of the number of phages present in each stock compared to the DAO method (Fig. 3). For each freshly prepared phage stock, a constant ratio between the number of phage genomes determined by qPCR and the phage number by means of DAO was obtained. This ratio in concentration between both methods (qPCR/DAO ratio) was phage dependent (Table 2). Figure 4 indicates that both methods showed low variability in quantification (C_v_ % < 20%), with qPCR in general being the most reproducible method (C_v_ % < 5%).

**Table 2:** Overview of the estimated number of phages detected with both methods, and the mean qPCR/DAO ratio. The concentration per stock of each phage was obtained by taking the median of four (qPCR) or three (DAO) technical replicates of the three stocks (1, 2 and 3).

Phage	Stocks	qPCR	DAO	Mean qPCR/DAO ratio (SD)
		Median genomes/ml	Median pfu/ml	
Acibel004	1	1,68E + 11	1,82E + 10	9.40 (±3.83)
	2	1,65E + 11	1,24E + 10	
	3	1,30E + 11	2,30E + 10	
Acibel007	1	3,20E + 11	6,10E + 09	67.41 (±20.40)
	2	3,37E + 11	5,70E + 09	
	3	9,79E + 11	1,08E + 10	
14/1	1	2,32E + 12	9,60E + 11	2.29 (±0.12)
	2	2,31E + 12	1,06E + 12	
	3	2,07E + 12	9,10E + 11	
PNM	1	3,27E + 12	1,57E + 12	2.20 (±0.27)
	2	3,08E + 12	1,54E + 12	
	3	3,88E + 12	1,55E + 12	
ISP	1	1,47E + 12	1,96E + 11	7.27 (±0.21)
	2	2,52E + 12	3,49E + 11	
	3	1,27E + 12	1,79E + 11

**Figure 5:**
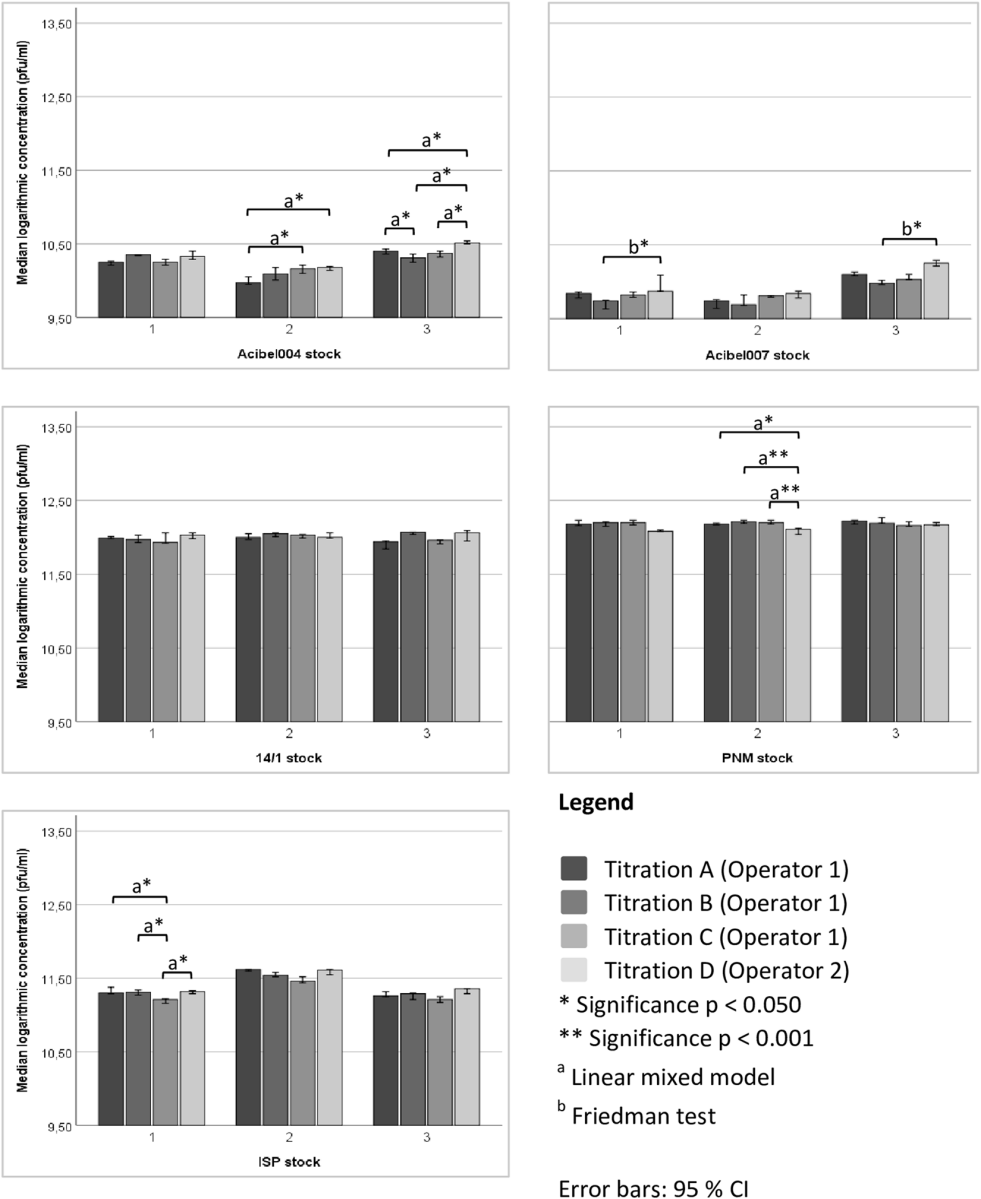
Intra- and inter-operator variability of quantification of both operators with DAO. SPSS statistical analysis was done using Linear mixed model (a) or Friedman test (b). Data presented as median logarithmic values with 95% CI.

The DAO method showed intra-operator variability as well as inter-operator variability (Fig. 5). Analysis of data operator 1 showed that for Acibel004 stock 2 (titration A and C, p = 0.007), Acibel004 stock 3 (titration A and B, p = 0.032) and ISP stock 1 (titration A and C, p = 0.001; titration B and C, p = 0.004) significantly different concentrations were obtained. Inter-operator variability, between operator 1 (titrations A-C) and operator 2 (titration D) was still more frequent: Acibel004 stock 2 (titration A and D, p = 0.004), Acibel004 stock 3 (titration A and D, p = 0.004; titration B and D, p < 0.001; titration C and D, p = 0.001), Acibel007 stock 1 and 3 (titration B and D, p = 0.027), PNM stock 2 (titration A and D, p = 0.002; titration B and D, C and D, p < 0.001) and ISP stock 1 (titration C and D, p = 0.002).

**Figure 6:**
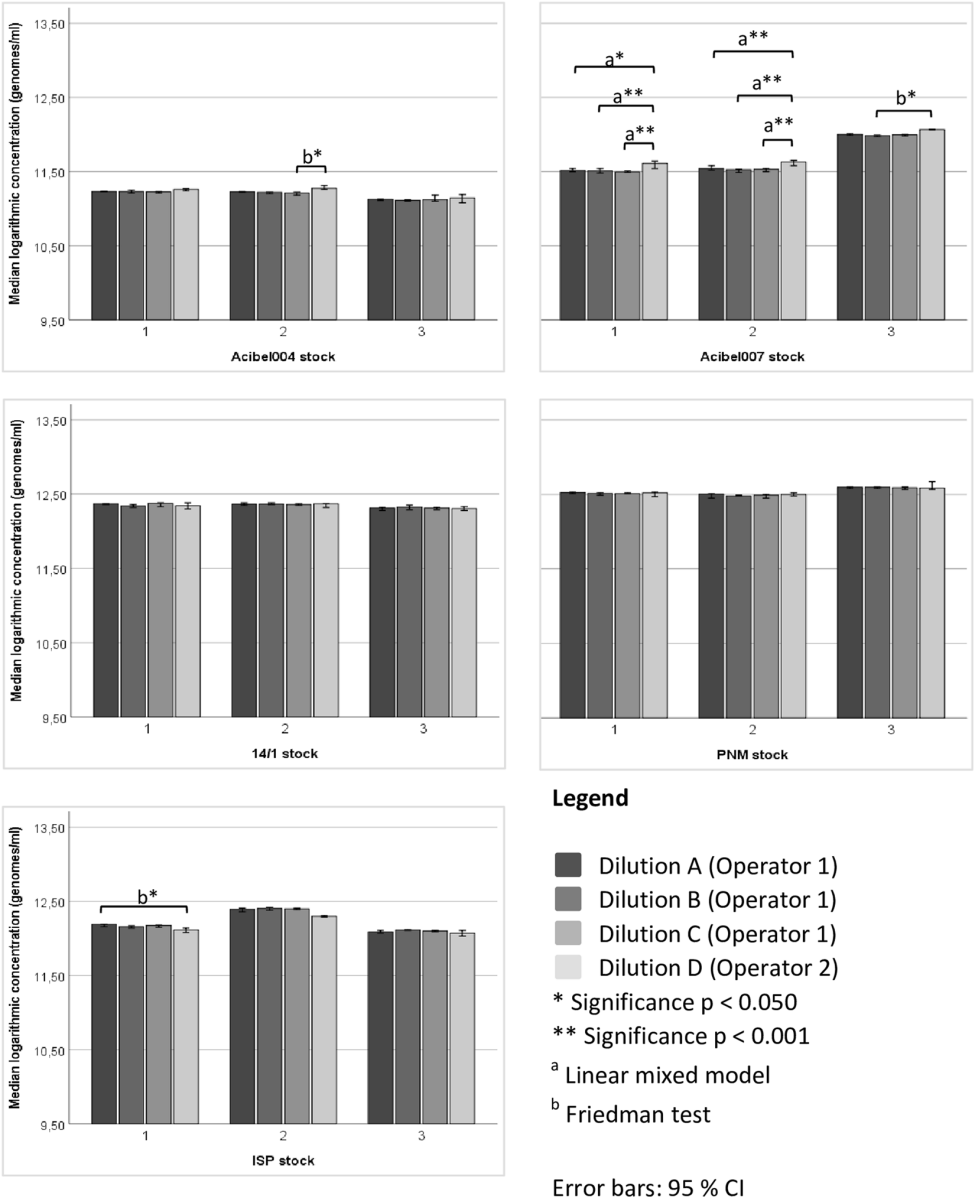
Intra- and inter-operator variability of quantification of both operators with qPCR. SPSS statistical analysis was done using Linear mixed model (a) or Friedman test (b). Data presented as median logarithmic values with 95% CI.

In contrast to the DAO method, qPCR data of both operators indicated no intra-operator variability (Fig. 6). But for some phages an inter-operator variability was observed, namely in Acibel004 stock 2 (dilution C and D, p = 0.037), Acibel007 stock 1 (dilution A and D, p = 0.001; dilution B and D, and C and D, p < 0.001), Acibel007 stock 2 (dilution A and D, B and D, and C and D, p < 0.001), Acibel007 stock 3 (dilution B and D, p = 0.016) and ISP stock 1 (dilution A and D, p = 0.006).

## Discussion

We describe the development of a qPCR platform for the molecular quantification of a mixture of five phages present in our model cocktail “bacteriophage cocktail 2” (BFC2) and compare its performance for freshly prepared stocks with the standard culture-based double agar overlay method (DAO), with regard to quantification, applicability and reproducibility.

For each phage, one primer pair was selected that was specific for detection, identification and quantification at a standardized annealing temperature of 60 °C and in a standardized amplification mixture with a MgCl_2_ concentration of 3.0 mM and a primer concentration of 0.2 µM. Quantification of the phages was possible by means of qPCR directly, *i.e*., without prior DNA extraction, on a freshly prepared crude phage stock in saline, on the premise that the stock was first diluted a hundredfold in HPLC-grade water, in order to avoid PCR inhibition by the NaCl in the saline, needed to store the phage cocktail in a stable manner.

When comparing quantification obtained with qPCR vs. DAO, the concentration of phages determined by qPCR was significantly higher for all five phages, compared with the measurements using DAO.

Furthermore, we observed a phage-dependent qPCR/DAO ratio which seems to be rather constant for each phage when analyzing different (freshly prepared) stocks of these phages. This ratio, determined on the basis of three (DAO) or four (qPCR) technical replicates of each of three biological replicates (different stocks), was 9.31 ± 3.80 for Acibel004, 66.29 ± 18.07 for Acibel007, 2.24 ± 0.12 for 14/1, 2.16 ± 0.26 for PNM and 7.28 ± 0.36 for ISP. These ratios are unrelated to phage particle morphology or genome size. Although both *P. aeruginosa* phages have the lowest ratio, there seems to be no similarity with regard to ratio for the *A. baumannii* phages. Given the apparent reproducibility and phage specificity of these ratios, the number of phage genomes in a freshly prepared stock, as determined by qPCR, might be converted into number of plaque forming units, after the ratio has been determined once initially for each new phage.

These differences can be explained by the fact that both methods are based on different principles to quantify the phages. DAO depends on detecting phages that are actively infecting the host, while qPCR is based on the detection of phage genome DNA, which comprises DNA from active and non-active phages, including free phage DNA from degraded virion particles.

Since we used freshly prepared phage stocks, which can be assumed to consist primarily of intact, infective phages, it is unlikely that qPCR overestimated the number of infective phages. Still, it is possible that during the centrifugation and filtration steps, part of the phages are already impaired, even in freshly prepared stocks, leading to overestimation of the number of infective phages by DNA-based techniques. Still, the fact that for each phage a different and rather constant ratio between qPCR and DAO is observed, does not seem to support random damage of phages during preparation as an explanation for the difference between both methods.

On the other hand, the difference between both methods may result from underestimation of the number of phages by means of the DAO method. This might be because multiple phage particles are responsible for the formation of only a single plaque or because only one out of more phages is able to establish infection in each infection cycle. Indeed, in a very detailed study, Gallet *et al*.^20^ showed how different characteristics of the phage infectious cycle, such as adsorption rate, lysis time (latency period) and burst size all influence plaque size, plaque productivity and average phage concentration in a plaque.

There are several advantages of qPCR over DAO, although DAO is assumed to provide an indication of the number of infective phage particles, whereas qPCR only provides information on the number of phage genomes, which might represent an important bias of the qPCR method. Subsequently, in order to develop a qPCR assay, primers need to be designed and thus it is necessary to determine the phage genome sequence in advance. However, phage genome sequencing is generally agreed to be mandatory for therapeutic phages to ascertain that the phages are virulent and free of bacterial virulence genes and antibiotic resistance genes^21^. With qPCR, quantification of the phages is accomplished in less than 3 hours in contrast to two days for the DAO method. Moreover, the qPCR method is less laborious as it requires far less handling and pipetting in comparison to the DAO method. Another drawback of the DAO method, adding to its laboriousness, is that three different titrations, with three different bacterial hosts need to be performed to assess the quantity of the five phages in BFC2. In order to be able to apply DAO, a specific match of bacterial host and phage must be met, whereas qPCR has no need of a bacterial host for an accurate quantification. In addition, intra- and interoperator reproducibility was higher for qPCR than for DAO. Also, for phages such as Acibel004 and Acibel007, *i.e*., for phages with indistinguishable plaque morphology, it is impossible to determine their individual abundance after mixing them in BFC2 and after application of BFC2 in a patient, whereas phages with similar plaque morphology can be easily quantified using the qPCR method, based on the specificity of the primers and the specific melting curve temperatures. The applicability of DAO in patient samples is further limited by the fact that, unlike phage suspensions, most clinical samples cannot be homogeneously mixed easily with the bacterial suspension and that moreover the sample might cause inhibition of the growth of the bacterial host, *e.g*. due to the antibiotics still present.

Although qPCR is measuring genomes and not (necessarily) active phage particles, and as such might overestimate the number of phages present, it still might provide with a valuable estimate of infective phages in the clinical sample as well. When it comes to the study of viscous clinical samples, such as tissues or sputa, it is difficult to isolate active phages. Complex sample processing procedures can affect the structure of the phages, e.g. loss of fragile tail fibers or of the tail itself, and thus influence the activity of the phages negatively. The loss in active phages will result in a lower amount of plaques obtained with DAO. Moreover, it can be assumed that the phages present in a clinical sample will be mostly those that are still actively infecting bacterial cells, and thus that the phages will be attached to or residing inside bacterial cells, since in a clinical environment non-infective phage particles outside bacterial cells may be quickly removed/degraded and will be outnumbered by intracellular phage genomes and particles. Therefore, it can be hypothesized that qPCR will quantify primordially phage DNA that is extracted from active phages associated with bacterial cells. Furthermore, the possible problem of overestimation in clinical samples of the number of infective phages by means of qPCR, due to amplification of free phage DNA from degraded phage particles, might be tackled by rendering free DNA not-amplifiable, e.g. by pretreatment of the sample with propidium monoazide prior to DNA extraction^22^ or by pre-treatment with deoxyribonucleases. Still, phage genomes in inactive, but intact phage heads will be amplified and may cause a certain degree of overestimation.

For quantification of stored phage stocks, qPCR seems to be not suited. If phages are stored under non-optimal conditions and low concentrations, infectivity can quickly and drastically drop, while DNA content in the vial will remain largely the same, due to DNA stability, which will result in overestimation of the number of infective phages by qPCR. In this case, the DAO method may be preferable and qPCR should be used only to determine the number of phages in freshly prepared stocks.

Although the DAO approach is less suited for enumeration of phages in patient samples, it may provide a more accurate estimate of the infectivity of the phage for the patient strain, since phage infectivity depends largely on the host strain, and since the patient bacterial strain is usually different from the bacterial strain that was used to propagate the phage during preparation of the phage stock.

On the other hand, this characteristic can also be seen as a drawback, since determining phage infectivity of the phage stock on the basis of phage infectivity of the production strain, may sometimes be of limited relevance for predicting its activity against the patient strain.

In conclusion, we made an elaborate comparison of the molecular qPCR method and the culture-based DAO method. Hence we set forward the abovementioned comparison as a preliminary guideline to take into account when using qPCR and DAO. Based on our analysis, we propose that qPCR is a valid alternative for the rapid and reproducible quantification of infective phages in freshly prepared phage stocks, that it seems possible to convert the obtained number of phage genomes into plaque forming units after initial establishment of a correction factor by comparison with the quantification obtained by means of DAO. Although a golden standard for phage quantification is hard to establish and depends on the application, our data suggest that a qPCR approach is a valid alternative to the current DAO method and might represent a valuable method of phage quantification in clinical samples.

## Supplementary information

Supplementary table (S1) and figures (S1-S3)

## Data Availability

All relevant data are within the paper.
