# Supplementary material for: Development of a qPCR platform for quantification of the five bacteriophages within bacteriophage cocktail 2 (BFC2)
Source: Sci Rep. 2019 Sep 25;9:13893. doi: 10.1038/s41598-019-50461-0 (PMC6761158; doi:10.1038/s41598-019-50461-0)
Supplement: Supplementary file 1 — Supplementary table (S1) and figures (S1-S3) [file 41598_2019_50461_MOESM1_ESM.pdf]

Supplementary information

Development of a qPCR platform for quantification of the five bacteriophages within bacteriophage cocktail 2 (BFC2)

Hans Duyvejonck[1,2], Maya Merabishvili[1,3,4], Jean-Paul Pirnay[3] , Daniel De Vos[3], Gilbert Verbeken[3], Jonas Van Belleghem[1], Tessa Gryp[1], Julie De Leenheer[2], Kelly Van der Borght[2], Leen Van Simaey[1], Stefan Vermeulen[2], Els Van Mechelen[2], Mario Vaneechoutte[1]

[1] Laboratory Bacteriology Research (LBR), Department of Diagnostic Sciences, Faculty of Medicine and Health Sciences, University of Ghent, Corneel Heymanslaan 10, 9000 Ghent, Belgium, [2] Department of Biosciences, Faculty of Education, Health and Social Work, University College Ghent, Keramiekstraat 80, 9000 Ghent, Belgium, [3] Laboratory for Molecular and Cellular Technology (LabMCT), Burn Wound Center, Queen Astrid Military Hospital, Bruynstraat 1, 1120 Brussels, [4] The Eliava Institute of Bacteriophages, Microbiology and Virology, Gotua 3, Tbilisi 0160, Georgia

Supplementary Table S1. Overview of the in silico designed primer pairs for the five BFC2 phages.

BFC2 phage	Primer		Sequence (5' → 3')	Melting temperature (°C)	Amplicon length (bp)	Amplicon Tm (°C)
Acibel004	1	F	GGCTGAACGTGTTCGTCAAC	60.0	133	
		R	CACCGAAGCGTGGGAAGTA	59.7		
	2	F	ATGGCTGAACGTGTTCGTCAAC	61.9	135	
		R	CACCGAAGCGTGGGAAGTA	59.7		
	3 *	F	GTATCGTCGGCTGTCGTGAA	60.2	113	80.88
		R	CGATCCTTCGTGGCGATCAT	59.9		
	4	F	GGCTGTCGTGAAAACGATA	55.7	101	
		R	CCTTCGTGGCGATCATAA D12	54.5		
Acibel007	1 *	F	TGTCGCTGAACATGGCGATA	59.5	132	83.46
		R	TCGTTAGCACGGTCAAGCA	59.6		
	2	F	GCTGAACATGGCGATACAA	56.0	125	
		R	TTAGCACGGTCAAGCATAC	55.3		
14/1	1	F	AGCCAGAGCGACGATATCAC	59.7	108	
		R	TTCGATTCCGCCATCACCAA	60.0		
	2	F	GCGACGATATCACCATCCAA	57.9	95	
		R	TCCGCCATCACCAATACTCG	59.9		
	3 *	F	AGCGATGGGTATCGGCAAAG	60.5	114	84.33
		R	TGGGCATTACCGAGGTTGAC	60.0		
	4	F	AATAGCGATGGGTATCGGCA	59.0	114	
		R	CCTGGGCATTACCGAGGTTG	60.8		
	5	F	TCGTTCAACGGCAAGTCGTA	60.0	130	
		R	AGCTCGACAAGCCAGATTCA	59.4		
	6	F	TCAACGGCAAGTCGTACAGC	60.9	128	
		R	GCAGCTCGACAAGCCAGATT	61.0		
	7	F	GGAATCCGCATCCAGTGCTA	59.9	100	
		R	CCCACTCGACGAACTTGACA	60.0		
	8	F	TCCGCATCCAGTGCTATACC	59.3	92	
		R	CTCGACGAACTTGACAAACG	57.5		
PNM	1 *	F	GGCGGACCGGAATAACAAGA	60.1	75	85.34
		R	CCGACCTCGACCAGTTGTG	60.4		
	2	F	AAGCTGGCGGACCGGAATAA	61.9	68	
		R	AGTTGTGCCAAGCCCTGCT	62.4		
ISP	1	F	GGATGGGGAACGCAATACCA	60.1	128	
		R	TCACTGCCACCCATTTGAGTA	59.3		
	2	F	GGAACGCAATACCAAGGTCTTG	59.8	121	
		R	ACTGCCACCCATTTGAGTAGC	60.6		
	3	F	GGGAACGCAATACCAAGGTCTTG	64.6	122	
		R	CACTGCCACCCATTTGAGTAGCT	64.6		
	4	F	AGCAGGTGGAAGTGGCATAG	59.8	147	
		R	CCTATTCCTCCGCCGATAGC	59.8		

5	F	GGTGGAAGTGGCATAGGGAAA	60.0	138	
	R	TCCTCCGCCGATAGCTTTAC	59.3		
6 *	F	CCGGCTTGACTCTCATTCCA	59.8	81	75.98
	R	AGCTACAACCGAGCAGTTAGA	58.8		
7	F	CTGTACCGGCTTGACTCTCA	59.1	94	
	R	CTTGAAAAAGCTACAACCGAGCAG	60.9		

*: Specific and efficient primer pair selected for further investigation; NA: Not applicable.

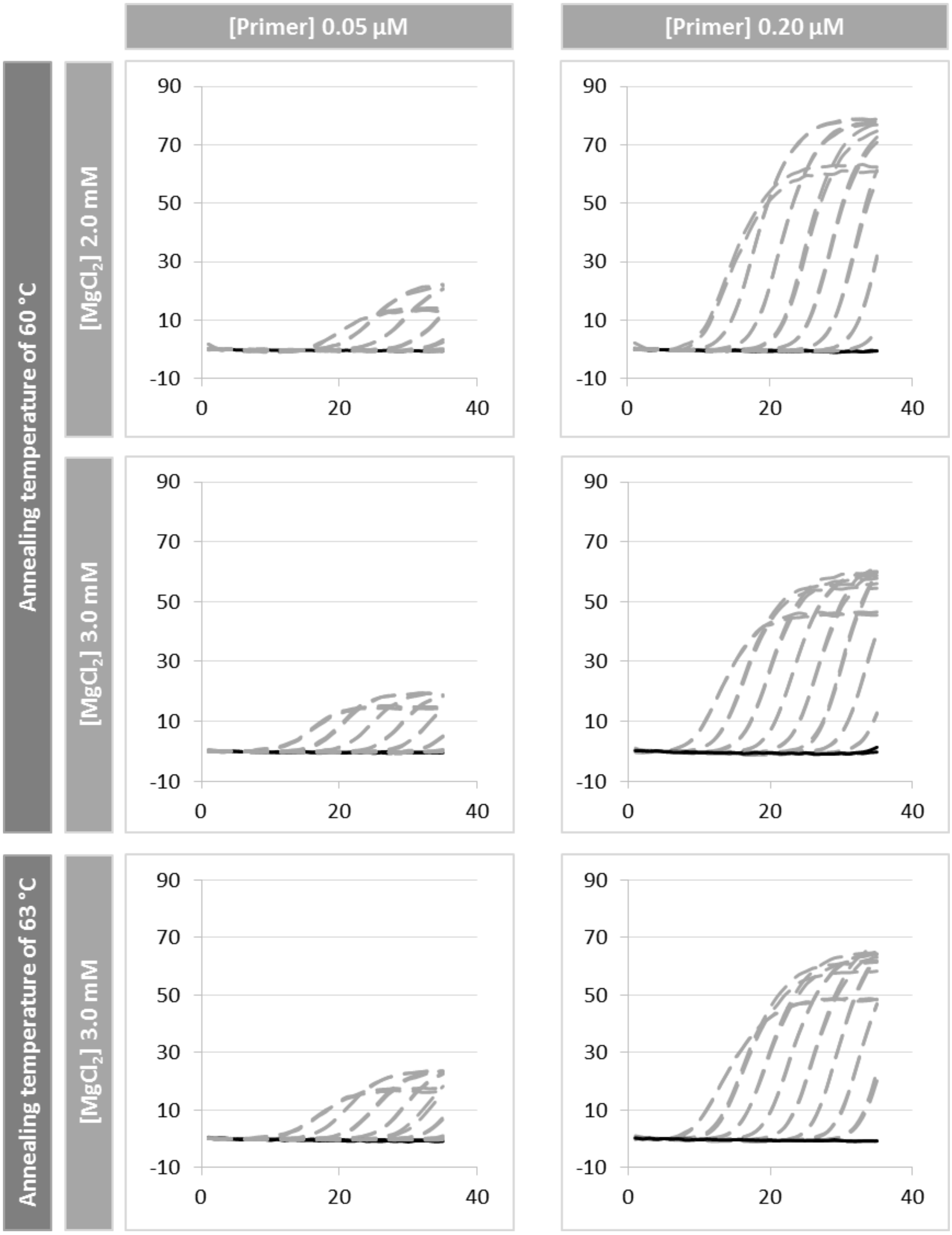

Supplementary Fig. S1. Influence of different annealing temperatures (60, 63 °C), $MgCl_2$ concentrations (2 and 3 mM) and primer concentrations (0.05 and 0.2 µM) on the efficiency of *in silico* designed primer pairs. Results for primer pair 1 of phage PNM are used for this graphic evaluation. The amplification curves of the ten-fold dilution series of the PNM phage (dotted curves), and water as a negative control (solid line), are being displayed.

X-axis: Cq values; Y-axis: Fluorescence intensity at 465-510 nm.

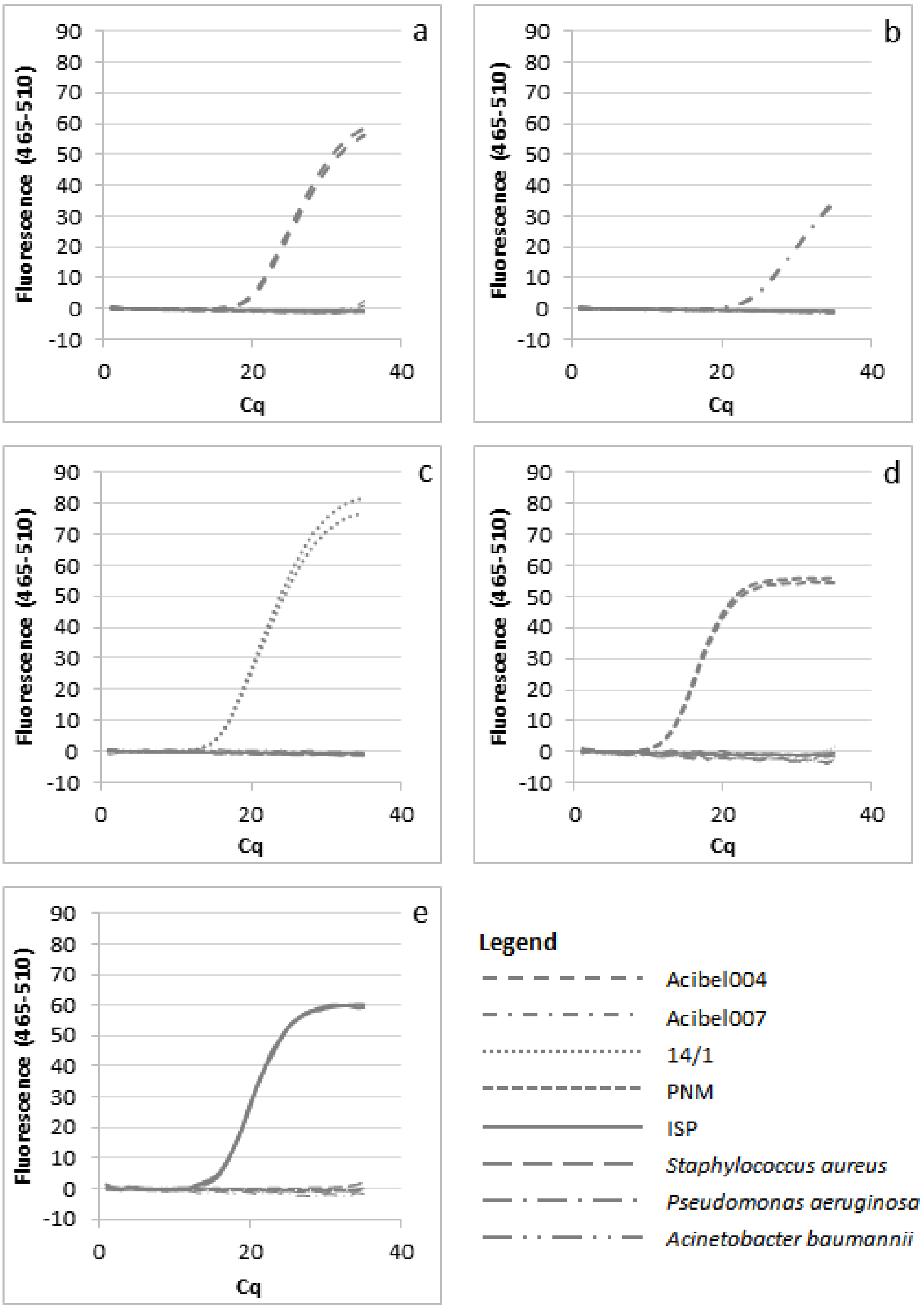

Supplementary Fig. S2. Evaluation of absence of cross reactivity for the primer pairs that were selected for each of the five phages. Primer pairs specific for Acibel004 phage (a), Acibel007 phage (b), 14/1 phage (c), PNM phage (d) and ISP phage (e) were tested against all five phages and the three bacterial hosts.

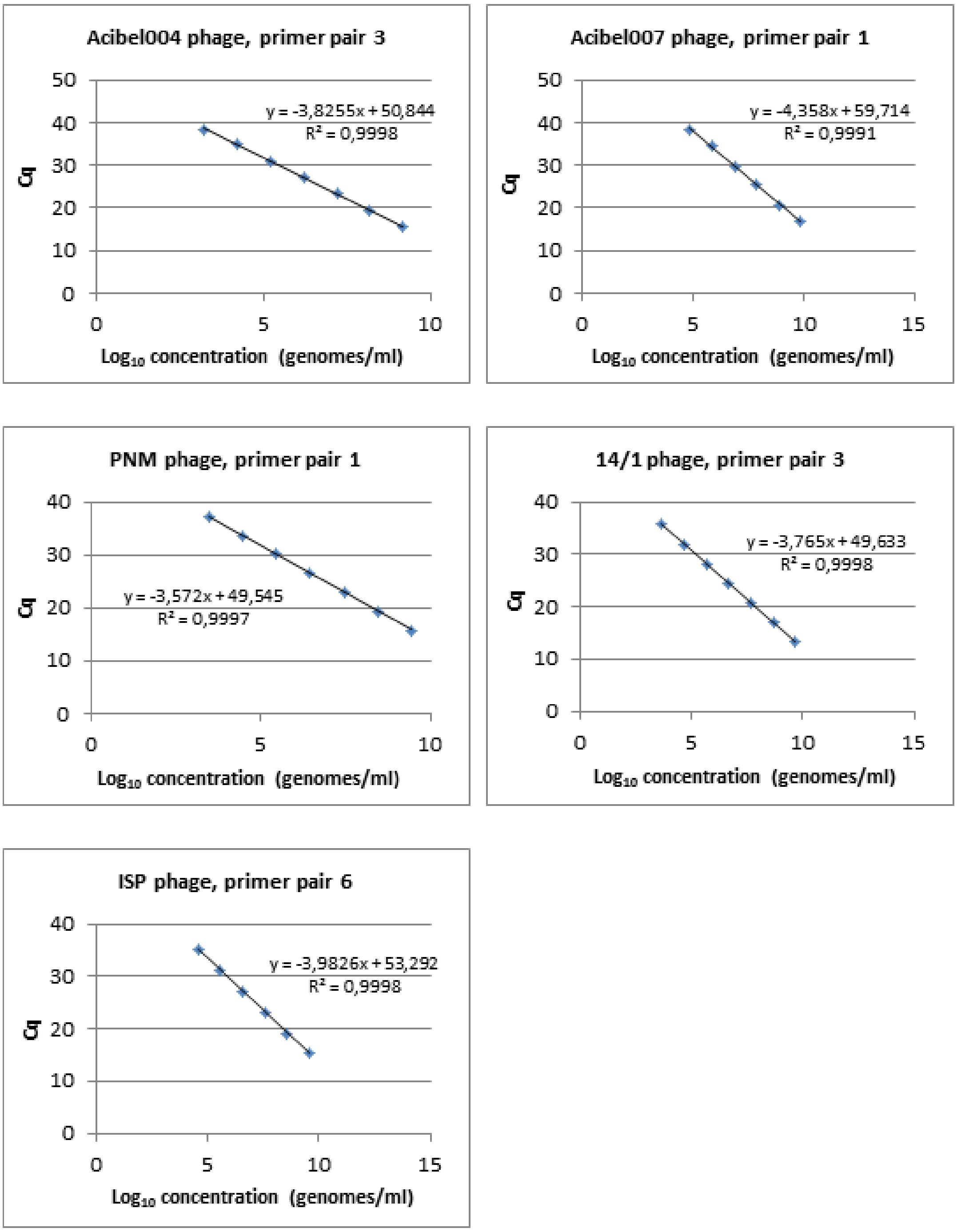

Supplementary Fig. S3. Calibration curves of all five phages.
